# Predicting retracted research: a dataset and machine learning approaches

**DOI:** 10.1186/s41073-025-00168-w

**Published:** 2025-06-11

**Authors:** Aaron H. A. Fletcher, Mark Stevenson

**Affiliations:** School of Computer Science, The University of Sheffield, Regent Court, Sheffield, S1 4DP UK

**Keywords:** Retraction prediction, Machine learning, Scientific publishing

## Abstract

**Background:**

Retractions undermine the scientific record’s reliability and can lead to the continued propagation of flawed research. This study aimed to (1) create a dataset aggregating retraction information with bibliographic metadata, (2) train and evaluate various machine learning approaches to predict article retractions, and (3) assess each feature’s contribution to feature-based classifier performance using ablation studies.

**Methods:**

An open-access dataset was developed by combining information from the Retraction Watch database and the OpenAlex API. Using a case-controlled design, retracted research articles were paired with non-retracted articles published in the same period. Traditional feature-based classifiers and models leveraging contextual language representations were then trained and evaluated. Model performance was assessed using accuracy, precision, recall, and the F1-score.

**Results:**

The Llama 3.2 base model achieved the highest overall accuracy. The Random Forest classifier achieved a precision of 0.687 for identifying non-retracted articles, while the Llama 3.2 base model reached a precision of 0.683 for identifying retracted articles. Traditional feature-based classifiers generally outperformed most contextual language models, except for the Llama 3.2 base model, which showed competitive performance across several metrics.

**Conclusions:**

Although no single model excelled across all metrics, our findings indicate that machine learning techniques can effectively support the identification of retracted research. These results provide a foundation for developing automated tools to assist publishers and reviewers in detecting potentially problematic publications. Further research should focus on refining these models and investigating additional features to improve predictive performance.

**Trial registration:**

Not applicable.

## Background

Retracting scientific articles is essential for safeguarding the integrity of the research record, but the growing number of retractions also reveals weaknesses in peer review and editorial oversight [1, 2]. Determining the extent of retractions is complicated by “stealth retractions”, which make the retracted articles difficult or impossible to trace, often involving the removal of the paper and the omission of a formal notice [3]. Separately, journals face a persistent tension between preventing the publication of flawed work and ensuring timely dissemination of results [4]. Although retracted research can still be useful-alerting the community to invalid findings or spurring new investigations-this utility depends on the clarity of its retracted status, which is often inconsistently handled. Unchecked, problematic work can damage authors’ reputations [5], tarnish journals [6], and undermine domain integrity [7].

The contemporary scientific publishing landscape further compounds the challenge of maintaining research integrity. Globally, the volume of research submissions escalates dramatically, straining the peer review system [8, 9]. This high-throughput environment necessitates an urgent need for supplementary tools. Automated screening methods, such as those explored here, could serve as valuable aids by flagging potentially problematic manuscripts early, thereby helping editors and reviewers focus their limited time on critical scientific assessments.

Once published, retracted papers can continue to influence discourse if their invalidation is overlooked. Avenell et al. demonstrated how just 12 misconduct-tainted clinical trials were repeatedly cited in systematic reviews and guidelines, substantially altering or obscuring conclusions [10]. Schneider et al. found that 96% of direct citations to a retracted 2008 clinical trial did not acknowledge its retraction [11], while Hsiao and Schneider showed that only 5.4% of citing contexts across 7,813 retracted papers reflected the retraction [12]. Even high-profile examples, such as the discredited vaccine-autism paper, accrue citations that rarely probe the retraction’s specific invalidations [13]. Although flawed data do not generally spread through secondary citations [14], the persistence of direct citations underscores the need for consistent, visible retraction notices. While recent advances, such as the CrossRef API directly integrating the Retraction Watch database into their metadata [15], more approaches are needed to increase the visibility of retracted works.

Retractions commonly stem from honest errors or misconduct such as fabrication, plagiarism, or falsified authorship, but the relative prevalence of each is disputed. For example, Steen [2] attributed 73.5% of PubMed retractions to errors, whereas Fang et al. [16] later, using additional sources, reclassified 15.9% of this dataset from errors to fraud, suggesting that clarifying a paper’s retraction status is more straightforward than classifying its cause.

Effectively identifying problematic research publications remains a significant challenge despite growing awareness of retraction harms and the importance of journal gatekeeping. Previous research has approached this issue from several angles: classifying the reasons why already withdrawn papers were retracted [17], analysing characteristics common among articles deemed to warrant retraction [18, 19], and highlighting systemic difficulties in retracting papers even when significant issues are known [20]. While these studies provide valuable insights into the nature and handling of flawed research, developing and evaluating predictive machine learning models designed to identify articles at high risk of future retraction prospectively has received limited investigation. This study aims to address this specific gap by (1) creating a dataset aggregating retraction information with bibliographic metadata, (2) training and evaluating various machine learning approaches to predict article retractions, and (3) assessing feature contributions using ablation studies.

## Methods

This study used existing data from open-access catalogues, databases, machine learning models and closed/open-sourced LLMs. The research design used was a retrospective observational study with a case-control approach.

### Dataset construction

Publicly available data from two online databases were used to construct the dataset (Retraction Watch and OpenAlex). Retraction Watch is a human-validated retraction dataset and is compiled from various sources, including journal databases, institutional reports, social media, and direct tips [21]. Although not exhaustive due to unannounced or “stealth” retractions, it provides partial metadata for some retracted articles, such as title, journal, publisher, and author. OpenAlex is an open-access online catalogue of academic publications, similar to Scopus and Web of Science, which aggregates data from multiple sources and releases monthly updates. These resources were combined to create a single dataset suitable for predicting article retractions [22].

A set of retracted articles were identified using the Retraction Watch dataset (Dataset downloaded on 24/07/2024). Only articles and review works were considered. Conference papers were excluded due to a mass retraction of conference papers undertaken by the Institute of Electrical and Electronic Engineers between 2009 and 2011 (having retracted over 10,000 such papers in the past two decades) [23] and because there is no process to retract papers from many conference venues. Retractions were limited to a 20 year period from 2000 to 2020 due to the lack of retracted works before this date, the median post-publication time to retraction being 1.8 years [24] and the increased use of natural language technologies subsequent to this period. Information from OpenAlex (API queried on 24/07/2024) was also used to filter out some works. A full list of exclusion criteria for journals and articles are shown in Tables 1 and 2.

**Table 1:** Journal exclusion criteria

Criteria	Description
CrossRef	If journal was not included in CrossRef’s journal title list.
Works Count	If work count (*based on OpenAlex API*) - total retraction count (*based on Retraction Watch Dataset*) < Sample Size (*1*)
Retraction Count	If journal total retractions < 5 (*determined by the Retraction Watch dataset*).

**Table 2:** Work exclusion criteria

Criteria	Description
Retracted Works	If Retraction Watch parameter *‘ArticleType’* not in {Research Article, Conference Abstract/Paper, Clinical Study, Review Article, Case Report, Meta-Analysis}
Retracted Works/Non-retracted works	If OpenAlex *‘source’* not in {Conference, Journal} and *‘type’* not in {article, review}
English Language	Work excluded if OpenAlex API *’language’* value not ’en’
ISSN Data	If OpenAlex API *‘issn’* value not available
OpenAlex ID	If OpenAlex API *‘id’* value not available
Article Type	If OpenAlex API *‘type’* value not in {article, review}
Publication Year	If *‘publication_year’* OpenAlex API value not available
Publication Year Minimum	If *‘publication_year’* OpenAlex API value < 2000
Publication Year Maximum	If *‘publication_year’* OpenAlex API value > 2020
Reformulated Abstract Length	If < 5 words
Unretracted works’s whose title contained	*“retraction”, “retraction:”, “withdrawn”, “correction”, “erratum”, “retracted”, “withdrawal”, “conclusion”, “editorial”, “contributions”, “commentary”, “contributors”*.
Retracted and unretracted works if abstract or title contained the words	*“elsevier”, “notice”, “editor”, “editors”, “publisher”*.

Another set of non-retracted articles was formed. For each retracted article, another article was randomly matched-pairs sampled from the same journal where a retraction had occurred, that was published in the same year as the retracted article, did not meet the works exclusion criteria outlined in Table 2, was not included in the retraction watch dataset and whose OpenAlex API flag of *‘is_retracted’* was False.

Articles containing keywords strongly indicating that it has been retracted were also excluded (e.g., “retraction”, “retracted”, “withdrawn”, “withdrawal”; full list: “retraction”, “retracted”, “retract”, “retractionwatch”, “retraction watch”, “removed”, “withdrawn”, “withdrawal”, “withdraw”, “retracted article”, “article”). This heuristic filtering risks excluding some valid non-retracted articles where keywords have alternative meanings (e.g., biological retraction), but was applied to reduce the inclusion of potentially mislabeled retracted articles in the control set.

The following features were extracted for each work (retracted and non-retracted): Abstract Inverted Index, Publication Date, Primary Topic, First Author, Institution, Citation Count, First Author Countries, Is Retracted Flag and Article Type.

Both sets (retracted articles and non-retracted articles) were combined and balanced through undersampling, resulting in a total of 9,028 pieces of research, with equal numbers of retracted and non-retracted. It was divided into training (64%), validation (16%) and test sets (20%). Group sizes were chosen with nested split using standard machine learning splits [25]. A balanced dataset was used, as the distribution of retracted works to non-retracted works across all works is highly imbalanced. This imbalance potentially leads to models learning a priori class distributions rather than learning from the features provided.

All textual fields were preprocessed by converted to lowercase, and eliminating non-ASCII characters, special punctuation, and numbers. The title and abstract were then combined. The Publication Date feature was converted solely to its year in YYYY format.

### Classifier creation

Multiple approaches to machine learning classification were trained: Feature-based and LLMs (Encoder-based and Decoder-based) classifiers.

Feature-based classifiers were selected that have demonstrated effectiveness for text-classification tasks: Gradient Boosting, SVM, XGBoost, Random Forest, MLP and Decision Trees [26]. A Super Learner model, an ensemble approach with multiple machine learning models, was also utilised. Several LLMs known for strong classification performance was selected. Contrasting encoder-based pre-trained models were included: BERT (“bert-base-uncased”), trained on a broad dataset, and BioBERT (“dmis-lab/biobert-base-cased-v1.2”), pre-trained on biomedical data. Contrasting decoder-based LLMs were selected, differentiated by their fine-tuning: Llama 3.2 (“unsloth/Llama-3.2-3B-bnb-4bit”), its instruction-tuned variant (“unsloth/Llama-3.2-3B-Instruct-bnb-4bit”), Gemma 2 (“unsloth/gemma-2-9b”), and its instruction-tuned variant (“unsloth/gemma-2-9b-it-bnb-4bit”). Unsloth’s versions were selected over the standard implementations because they significantly reduced VRAM requirements due to optimisations like 4-bit quantisation and optimised kernels while maintaining performance. This was crucial as fine-tuning the standard base models exceeded available hardware. Commercially available LLMs were also evaluated: GPT-4o mini [27] and Claude 3.5 sonnet [28]

**Fig. 1:**
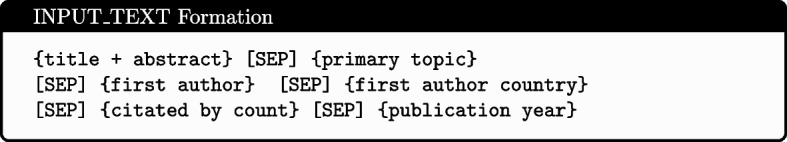
Input format for encoder-based models

**Fig. 2:**
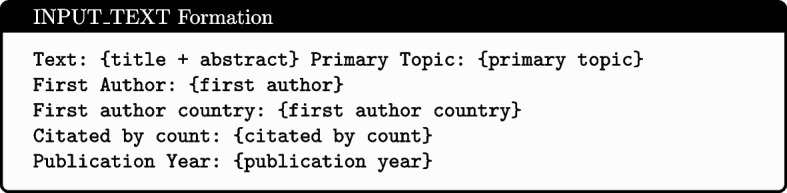
Input format for decoder-based models

**Fig. 3:**
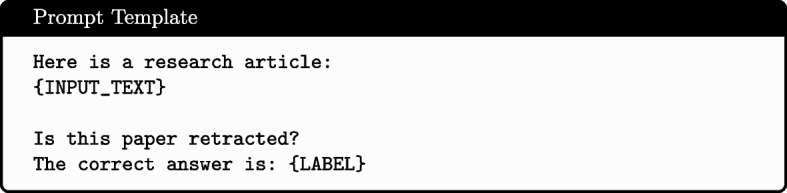
Prompt template used for structuring input data during model fine-tuning

Different model architectures necessitated different input formats. Inputs for feature-based classifiers were formed into a single vector representation: Numerical features (Publication Year and Citation Count) were min-max normalised to scale between 0 and 1. Categorical features (First Author’s country) were one-hot encoded based on the training data subset. All other text features were represented using term weights produced by Best Matching 25 [29] with $k = 2$, $b = 0.3$ and no maximum vocabulary length specified. All features were then concatenated to produce the single vector representation. Inputs for the encoder-based LLM models consisted of the features (Text, Primary Topic, First Author, First Author Country, Citated By Count and Publication Year) concatenated with [SEP] tokens separating each feature - as shown in Fig. 1. Decoder-based LLMs used the prompt template illustrated in Figs. 2 and 3.

All models were trained using the training dataset, with early stopping being determined using the validation dataset to prevent overfitting. An expectation of this was the commercial models (i.e. GPT-4o mini and Claude 3.5 sonnet), where a zero-shot approach was used. LLM fine-tuning was conducted using supervised fine-tuning for a maximum of 10 epochs. Only the pooling layer and the classification head were updated with encoder-based models. Decoder-based LLMs were fine-tuned using the input format and prompt template illustrated in Figs. 2 and 3. With these, a low-order rank adaptation approach was used, with output vocabulary restricted to “yes” and “no” tokens. During testing, these models were evaluated by providing the input text and the question without the label to assess their ability to predict the retraction status independently, with a softmax of the logits for the “yes” and “no” output tokens forming the model’s prediction.

All models output a binary classification label; “0” denoting if a piece of work is retracted and “1” not. Models were evaluated using the testing dataset. From this, model performance was measured using standard metrics for classification problems. Accuracy is the proportion of instances correctly classified as either retracted or non-retracted. Precision, recall and F1 scores are computed individually for the retracted and non-retracted classes and then averaged.

### Ablation

To evaluate the relative contribution of each feature to classifier performance on all feature-based classifiers, an ablation study was performed. For each feature, an “ablated” version of the dataset was created by removing that feature from all training instances while leaving the remaining features intact. Every feature-based classifier was then re-trained from scratch using the ablated dataset, and evaluated on the same test set employed for the full-featured models.

For instance, when ablating the *Abstract* feature, all tokens originating from the abstract were excluded, but the *Title*, *Primary Topic*, *Publication Year*, *First Author*, *First Author’s Country*, and *Citation Count* features were retrained. This procedure was repeated for each of the remaining features in turn. The resulting evaluation metrics (accuracy, precision, recall, and F1) were then compared against the “full-feature” baselines to quantify the performance drop caused by removing that feature. Lower scores in the ablated setting indicate a more critical feature, as its removal impairs model performance more severely.

## Results

### Dataset characteristics

**Fig. 4:**
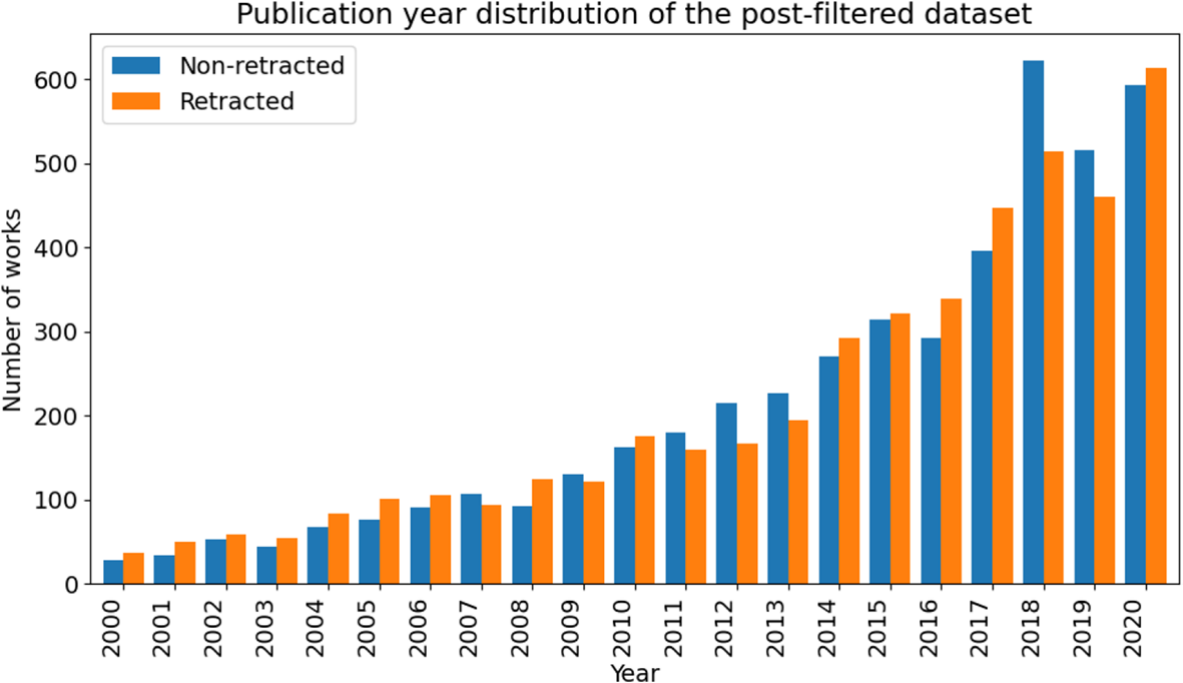
Publication year distribution for retracted and non-retracted works

Each year’s works distribution is shown in Fig. 4. It can be seen that the number of included works associated with each year increases over time, reflecting the trend of retractions increasing over time in the original Retraction Watch dataset.

In the generated dataset, 7.54% of the articles reported as retracted in Retraction Watch were not marked as retracted by OpenAlex, possibly because OpenAlex’s metadata is derived from multiple input sources. This discrepancy further illustrates the difficulty of identifying retracted research since it may not be labelled as such. This discrepancy has been recently directly addressed with CrossRef integrating the Retraction Watch database into the metadata returned from their API.

Analysis of correlations between journal features revealed two notable findings:

1. A weak, significant positive correlation between the work count log and the retraction count log (Pearson correlation coefficient 0.065, p-value < 0.05). This seems counterintuitive, as more retractions are likely to occur given more publications, and hence, a strong positive correlation would be present. This finding could indicate that journals that publish fewer works are less proactive at detecting potential retractions, that publishing research that will be retracted is more complicated within journals with greater work output, presumably due to increased scrutiny of these works, or that journals that publish less spend more time on the peer review process and manuscript handling, so retractions are not as necessary.
2. A strong negative correlation was observed between the journal retraction count and the log of the h-index (Pearson correlation coefficient −0.656, p-value < 0.05) in this dataset. Interpreting this statistical association requires caution. While the h-index reflects a journal’s citation impact, assuming this directly translates to more rigorous peer review or higher quality submissions in a way that consistently prevents retractions is an oversimplification and lacks an evidence-based cause. Factors such as publisher policies, editorial vigilance in post-publication monitoring, and specific decisions on handling potential misconduct determine the number of recorded retractions. These practices can vary considerably between journals, potentially independent of their h-index. Therefore, the observed correlation likely reflects a complex interplay of factors rather than a straightforward link between impact factor and retraction necessity.

### Classifier performance

Results for all classifiers are presented in Table 3, showing performance for both the retracted and not retracted classes. The highest-scoring approaches for each metric are highlighted in bold. Commercial models were excluded from further analysis as all commercial models responded that no research was retracted within the testing dataset. This was thought to be due to the safety restrictions implemented within these models, which prevented responses that could be considered problematic [30].

**Table 3:** Retraction classifier performance results

Model	Acc.	Non-Retracted			Retracted		
		P	R	F1	P	R	F1
Logistic Regression	0.638	0.638	0.647	0.642	0.639	0.630	0.635
Decision Tree	0.568	0.570	0.574	0.572	0.568	0.564	0.566
Random Forest	0.666	0.648	**0.731**	**0.687**	0.689	0.601	0.642
SVM	0.671	0.655	0.725	0.688	**0.690**	0.616	0.651
XGBoost	0.665	0.654	0.705	0.679	0.678	0.624	0.650
AdaBoost	0.631	0.619	0.684	0.650	0.645	0.577	0.609
Super Learner	0.669	0.661	0.699	0.680	0.678	0.640	0.659
MLP	0.655	0.650	0.675	0.663	0.660	0.634	0.647
Gemma 2-base	0.553	0.615	0.292	0.396	0.534	0.816	0.645
Gemma 2-instruct	0.529	**0.730**	0.098	0.173	0.515	**0.963**	0.671
BERT	0.609	0.612	0.602	0.607	0.606	0.616	0.611
BioBERT	0.608	0.598	0.668	0.631	0.621	0.548	0.582
Llama 3.2-base	**0.682**	0.686	0.674	0.680	0.678	0.689	**0.683**
Llama 3.2-instruct	0.535	0.714	0.121	0.208	0.518	0.951	0.671

All models outperformed random guessing (i.e. 0.5 as this is a binary classification task), although the improvement varies considerably between models. The highest accuracy (0.682) is achieved by Llama 3.2-base, although accuracy scores overall are generally higher for more traditional feature-based approaches such as gradient boost, SVM, XGBoost, and Random Forest achieved superior precision compared to the more modern contextually aware LLMs.

Regarding the retracted class, SVM achieved the highest precision (0.690) and Llama 3.2-base the highest recall (0.683). Interestingly, both instruction-tuned decoder-based LLMs (Gemma 2-instruct and Llama 3.2-instruct) also achieve high recall for the retracted class but this is achieved by predicting retracted for the majority of instances, as demonstrated by the very low recall for the non-retracted class. This could be due to instruction tuning, as they are trained to be more cautious and risk-averse, indicating that instruction-tuned models might not be suitable for this type of classification task.

These findings establish baseline results using the dataset.

### Ablation analysis

The importance of individual features to the feature-based classification models was explored by conducting an ablation study on all input features. Datasets were created for each feature by permuting the data to exclude that feature and then averaging the evaluation metrics (F1 score, precision, recall, accuracy) across all models for each ablation. Lower scoring metrics indicate a greater contribution to the performance of a classifier.

Several observations on the ablation of features can be made given the results reported in Table 4.

**Table 4:** Ablation performance metrics: lowest scoring ablations are in bold

Model	Acc.	Non-Retracted			Retracted		
		P	R	F1	P	R	F1
Abstract	0.655	0.670	0.612	0.638	0.645	0.678	0.669
Citation Count	0.648	0.659	0.611	0.634	0.638	0.684	0.660
First Author	0.649	0.662	0.609	0.634	0.639	0.689	0.663
First Author Countries	0.649	0.663	0.604	0.632	0.638	0.693	0.664
Primary Topic	0.644	0.658	0.602	0.628	0.634	0.687	0.659
Publication Year	**0.641**	**0.656**	**0.594**	**0.622**	**0.630**	**0.688**	**0.657**
Title	0.648	0.657	0.618	0.636	0.641	0.678	0.658

The publication year proved to be the most crucial feature, with its ablation resulting in the lowest scores across all metrics. This finding is interpreted not as implying that specific calendar years inherently produce riskier research but rather as indicating that the model effectively learned temporal trends in retraction patterns present within the 2000-2020 study period. This aligns with the significant increase in overall publication volume and the corresponding absolute number of retractions observed over these two decades. The temporal strength’s dominance in the ablation study underscores the challenge that its powerful signal might eclipse other valuable predictive features, highlighting the importance of identifying additional robust signals beyond publication date alone. The Primary Topic feature also demonstrated substantial importance, producing the second-lowest scores when ablated. Reduction in performance when First Author Countries are ablated indicates the likelihood that a work will be retracted, supporting previous findings [31].

Contrary to what might be intuitively expected, the abstract, despite being the longest and most detailed textual component, emerged as the least influential feature across all evaluation metrics. When ablated, it yielded the highest average scores for accuracy (0.655), precision (0.657), recall (0.655), and F1 score (0.654), indicating its removal had the least negative impact on model performance. This counterintuitive finding regarding the abstract’s limited influence could be attributed to several factors. First, structured metadata features (like publication date and primary topic) may provide more consistent and unambiguous signals for classification compared to the potentially noisy and variable nature of abstract text. Second, there might be considerable information redundancy between the abstract and other textual features like the title, making its individual contribution less distinctive.

## Discussion

Machine learning approaches show potential for identifying retracted papers using the created open-access dataset. While machine learning models trained on the dataset outperformed random guessing in identifying retracted papers, their overall performance indicates significant challenges remain. The results suggest a reliance on correlational patterns within the features used (such as publication year and author country) rather than a deep understanding of research flaws.

One of the potential applications of the classifier described above is as a tool during the peer review process, in much the same way that text similarity tools are often used to identify potential plagiarism. The required level of precision or recall would depend on how the tools would be used. If used as a screening tool to flag potentially problematic papers for additional review, a high recall would be preferable to avoid missing articles that are subsequently retracted. However, if used as a check which a submitted article must pass then high precision would be necessary to avoid the suppression of valid research. The performance of the models reported above, while promising, indicates that identification of retracted articles is not a trivial prediction task and may not be sufficient for some purposes. The decision regarding the involvement of systems to detect potential retractions within the peer review process is ultimately the choice of publishers.

The automatic prediction of potential retractions also raises ethical concerns. Predictive models, such as the ones described here, can introduce bias thereby raising potential fairness issues [32]. Such biases can unfairly penalise the groups more likely to be identified as producing research that will be retracted (e.g., first authors from particular locations) while benefiting those it is less likely to identify. This could introduce inductive bias into investigations, potentially leading to unforeseen consequences in the scientific publishing landscape, such as influencing which research questions are investigated and which methodologies are applied. In addition, authors may attempt to report results in ways that avoid detection by these models, potentially leading to self-censorship or overly cautious reporting of results. Conversely, bad actors with knowledge of these models may exploit that information to avoid detection, potentially facilitating the dissemination of invalid results.

An important consideration is how best to apply these models in practice. While machine learning classifiers can highlight publications at higher risk of retraction, final decisions on whether a paper should be investigated or retracted must rest with human experts-editors, reviewers, and domain specialists. For example, automated models flag potential anomalies in medical and clinical contexts, but the ultimate judgment requires expert oversight [33, 34]. Similarly, the classifiers reported here are intended to aid decision-making rather than stand-alone arbiters of scientific validity. A fully automated retraction process is not desirable, nor is it necessarily the duty of model developers to initiate or recommend retraction investigations on every flagged paper. Instead, these outputs can be a starting point for further human-led scrutiny. This workflow ensures that any potential reasons for retraction-which may be multifaceted and not always captured by the model-are carefully examined. It also prevents the undue penalisation of authors, institutions, or countries that might otherwise be overrepresented due to biases in the training data. By maintaining a robust human-in-the-loop process, publishers and editorial boards can leverage model predictions ethically and effectively to uphold the reliability of the scientific record.

## Limitations

This study has several notable limitations. The study design relied on a single data source, the Retraction Watch database, which provides valuable but incomplete coverage. The dataset is heavily skewed towards English, as the source for non-retracted articles (OpenAlex) comprises 75% English publications [35]. The language distribution within the Retraction Watch dataset is not readily available. The presence of “stealth retractions”, wherein papers are removed without official notice or may not be reported to Retraction Watch, creates the potential for missed or under-detected retractions. Additionally, it was retrospective, using data from 2000 to 2020, which limits the ability to assess the models’ real-time or prospective effectiveness in detecting erroneous work at publication. Theoretical limitations exist within the model choice, as they capture correlational rather than causal relationships, potentially leading to false positives or negatives, as using these patterns can misrepresent the underlying reasons for retractions. Data was sampled from 2000 to 2020, which would not represent more recent changes in retracted works. Since 2020, there have been innovative natural language generation models that could potentially increase the count of retracted works. Features that are not fully representative of a piece of research were used. Due to copyright restrictions, abstracts and metadata were used rather than full-text articles. Indicators of methodological errors or unsupported conclusions might appear in the main text and not the title and abstract, potentially reducing the reliability of our retraction-prediction metrics.

Relatedly, models trained on historical data inherently struggle to identify novel misconduct methods absent from their training set. Consequently, as unethical actors develop new bypass techniques, these models lag in detecting them. While continuous dataset updates can help mitigate this delay, human involvement remains essential to identify novel threats as they emerge. A key potential advantage of any perfected automated approaches is their ability to consistently apply detection for existing issues at scale.

Additionally, some LLM may have been partly trained on the same corpus used to develop or validate our dataset, inflating their performance scores. This issue does not affect purely feature-based approaches but undermines the reliability of LLM-derived results. Features such as the first author’s country or institution may reflect systemic biases in scientific publishing rather than genuine predictors of flawed work. Such biases risk penalising authors from certain regions or affiliations if used in editorial decision-making. Models may overfit to spurious textual or demographic correlations in the training data, leading to unjustified flags or missed detections when applied to new, diverse datasets.

## Conclusions

This research demonstrates the potential of machine learning approaches in predicting retracted articles, contributing to efforts aimed at enhancing the integrity of scientific publication. By creating a novel open-source dataset that combines information from the Retraction Watch database and the OpenAlex API, a resource for future investigations in this area has been contributed. Our dataset encompasses 9,028 articles published between 2000 and 2020, evenly divided between retracted and non-retracted works, and includes a variety of features such as abstracts, citation metrics, and author information.

Experiments showed that, with the exception of the recently released Llama 3.2 base model, traditional feature-based classifiers, such as gradient boosting machines and SVMs, outperformed contextual language models like BERT, BioBERT, and Gemma in terms of precision. The best-performing model achieved a precision of 0.690, indicating that while machine learning techniques hold promise, there remains a need for significant improvement before they can be effectively integrated into the peer review process. The ablation study highlighted the importance of the publication year, primary topic and the first author’s country in predicting retractions in this dataset, aligning with previous findings that suggest certain demographics may be more prone to retractions due to various factors.

### Future work

There is potential for the approaches described here to be extended by making use of additional information with the potential to assist in the identification of retracted research. For example, the citation network of references to a paper and the references within the paper itself may provide useful information. In addition, the models described here analysed abstracts, but analysis of the full text itself could potentially allow models to evaluate flaws in methodology, result synthesis or false conclusions. Finally, analysis of the full author list of an article could reveal patterns of collaboration or even help to identify potential paper mills.

## Data Availability

The dataset supporting the conclusions of this article is available in the Predicting Article Retractions repository [36].

## Abbreviations

LLM: Large language model
NLP: Natural language processing
SVM: Support vector machine
MLP: Multilayer perceptron
XGBoost: eXtreme gradient boosting
BERT: Bidirectional encoder representations from transformers
Llama: Large language model meta AI
